# Design of New α-Conotoxins: From Computer Modeling to Synthesis of Potent Cholinergic Compounds

**DOI:** 10.3390/md9101698

**Published:** 2011-09-28

**Authors:** Igor E. Kasheverov, Maxim N. Zhmak, Alexey Y. Khruschov, Victor I. Tsetlin

**Affiliations:** Shemyakin-Ovchinnikov Institute of Bioorganic Chemistry, Russian Academy of Sciences, Miklukho-Maklaya Street, 16/10 Moscow 117997, Russia; E-Mails: zhmak@front.ru (M.N.Z.); khrushh@rambler.ru (A.Y.K.); vits@mx.ibch.ru (V.I.T.)

**Keywords:** α-conotoxin analogs, nicotinic acetylcholine receptors, acetylcholine-binding proteins, computer modeling, radioligand analysis

## Abstract

A series of 14 new analogs of α-conotoxin PnIA *Conus pennaceus* was synthesized and tested for binding to the human α7 nicotinic acetylcholine receptor (nAChR) and acetylcholine-binding proteins (AChBP) *Lymnaea stagnalis* and *Aplysia californica*. Based on computer modeling and the X-ray structure of the *A. californica* AChBP complex with the PnIA[A10L, D14K] analog [1], single and multiple amino acid substitutions were introduced in α-conotoxin PnIA aimed at compounds of higher affinity and selectivity. Three analogs, PnIA[L5H], PnIA[A10L, D14K] and PnIA[L5R, A10L, D14R], have high affinities for AChBPs or α7 nAChR, as found in competition with radioiodinated α-bungarotoxin. That is why we prepared radioiodinated derivatives of these α-conotoxins, demonstrated their specific binding and found that among the tested synthetic analogs, most had almost 10-fold higher affinity in competition with radioactive α-conotoxins as compared to competition with radioactive α-bungarotoxin. Thus, radioiodinated α-conotoxins are a more sensitive tool for checking the activity of novel α-conotoxins and other compounds quickly dissociating from the receptor complexes.

## 1. Introduction

α-Conotoxins are at present the most precise tools for research on nicotinic acetylcholine receptors (nAChRs), mainly due to their relatively high specificity for different nAChR subtypes and small size, enabling solid-phase synthesis of α-conotoxins in large amounts (see, for example, reviews [2–5]). Involvement of distinct nAChR subtypes in muscle dystrophies, psychiatric, and neurodegenerative diseases [6–8] makes practical application of different cholinergic ligands crucial, including α-conotoxins as selective markers of the respective subtypes in normal state and pathologies, as well as for designing new drugs. At present several α-conotoxins are at different stages of preclinical tests [9,10]. The search for new naturally occurring α-conotoxins and design of α-conotoxin analogs is very active as seen from our recent review [11].

Here we tried to make new α-conotoxin analogs of higher affinity and selectivity to a homooligomeric α7 nAChR. This subtype is one of the best presented, especially in the brain, and appears to play an important role in Alzheimer’s disease [12,13]. That is why there is a need in specific labeling and quantitative measurement of the α7 nAChR levels. Among α-conotoxins, for some time such a role was ascribed to α-conotoxin ImI [14,15] but later its binding to some other subtypes was registered [16,17]. Another candidate was α-conotoxin PnIA, a α3β2 nAChR blocker, where a single A10L mutation shifted its specificity in favor of α7 nAChR [18,19]. We took this peptide as a basis for designing new α-conotoxins acting on α7 nAChR.

To choose the amino acid substitutions, we relied upon computer modeling, based mainly on the X-ray structures of the *Aplysia californica* and *Lymnaea stagnalis* acetylcholine-binding proteins (AChBPs) and their complexes. These proteins are excellent structural models for the ligand-binding domains of all nAChR subtypes and pharmacologically are closest to α7 nAChR [20,21]. Since the X-ray structures of both these proteins are known, as well as the X-ray structures of *Aplysia californica* AChBP with three different α-conotoxins [1,22–24], it seemed to be a good system to design α-conotoxins with a higher selectivity. In view of this, the activity of the synthesized compounds was tested by radioligand analysis with human α7 nAChR in the GH_4_C_1_ cell line, and also with the AChBPs of the two species. Moreover, it was carried out not only in competition with the [^125^I]-α-bungarotoxin ([^125^I]-αBgt), but with the radioiodinated derivatives of our novel α-conotoxins.

## 2. Results and Discussion

### 2.1. Computer Modeling and Choice of Amino Acid Substitutions

Computer modeling of α-conotoxin PnIA analogs and their docking to AChBPs and nAChRs was performed using the X-ray structures of α-conotoxins PnIA and PnIB [25,26] and the techniques and programs earlier employed with different α-conotoxins [1,27,28] and α-neurotoxins [29]. Models of the two AChBPs in complexes with α-conotoxin PnIA analogs were built on the basis of the X-ray structure for *Aplysia californica* AChBP in complex with PnIA[A10L, D14K] [1], taking into account also the AChBP complexes with α-conotoxins ImI and TxIA[A10L] [22–24] and with α-cobratoxin [30]. For analysis of complexes with the α7 nAChR, the *Torpedo marmorata* nAChR electron microscopy structure [31] was used.

Docking allowed us to choose several substitutions in the α-conotoxin PnIA amino acid sequence which might increase the affinity and/or selectivity. A higher selectivity might result also due to a drop in binding to other targets, yet saving the original affinity towards a certain receptor subtype. These peptides are shown in Table 1. The largest changes could be expected on introduction of charged residues into positions 5, 7 and 14 making possible strong ionic interactions between α-conotoxins and the targets.

Interestingly, charged residues in positions 5 and 7 (D5 and R7) are present in all known α-conotoxins of the “4/3 subgroup” (α-conotoxins ImI, ImII and RgIA), which have certain affinity to several nAChR subtypes including α7 nAChR [14,15,17,32], as well as in α-conotoxin RgIA which is a highly specific antagonist of α9α10 nAChR [33,34]. Another α9α10 nAChR specific ligand—α-conotoxin Vc1.1 of the “4/7 subgroup”—also has D5 and R7 residues in its amino acid sequence [35]. On the other hand, a positively charged R5 is present in α-conotoxin TxIA, a potent blocker of α3β2 nAChR and efficient ligand of *Lymnaea stagnalis* AChBP [24]. Histidine in position 5 can be found in the sequences of several α-conotoxins (GIC, PeIA, OmIA) acting on different neuronal nAChRs [36–38]. The increase in the affinity due to incorporation of a positive charge into the *C*-terminal region has been demonstrated previously both for α-conotoxins targeting the muscle-type nAChRs [27] and for α-conotoxin PnIA interacting with neuronal nAChRs [1].

### 2.2. Preparation of Synthetic α-Conotoxin Analogs

**Figure 1:**
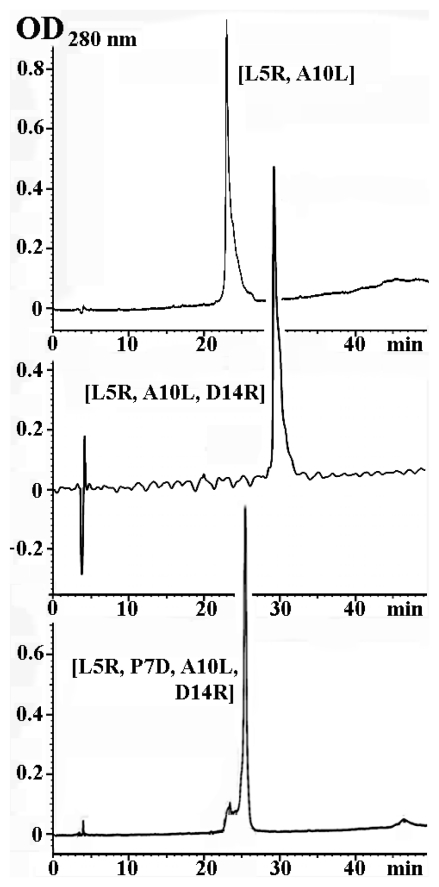
HPLC re-chromatography of selected α-conotoxin PnIA analogs.

Novel α-conotoxin analogs were prepared by the solid phase peptide synthesis, similarly to the earlier described syntheses of PnIA[A10L] and PnIA[A10L, D14K] [1]. The structures of all α-conotoxins purified by HPLC (see some in Figure 1) were confirmed by MALDI-TOF mass-spectrometry (see Table 1).

### 2.3. Binding Assays with α-Conotoxin Analogs

#### Competition Radioligand Assay with [^125^I]-Labeled α-Bungarotoxin

**Figure 2:**
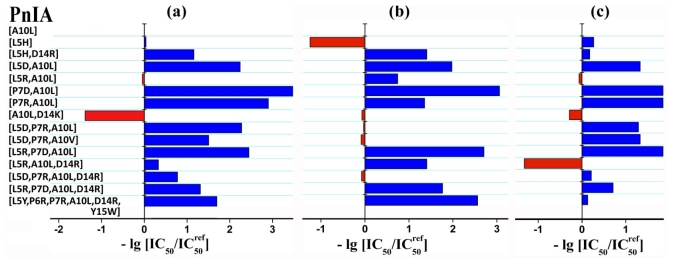
Bar presentation of the change in potency of each analog for (**a**) *L. stagnalis* AChBP, (**b**) *A. californica* AChBP, and (**c**) human α7 nAChR. This change was evaluated as logarithm of ratio of IC_50_ values for respective analog and PnIA[A10L]. A decrease in affinity to respective target for concrete analog as referred to PnIA[A10L] is represented in blue colored bars; and an increase—in red colored bars.

All the synthesized compounds were tested in competition with [^125^I]-labeled α-bungarotoxin ([^125^I]-αBgt) for binding to human α7 nAChR in the GH_4_C_1_ cell line and to AChBPs. The results are presented in Table 2 and are also depicted as a histogram (Figure 2). It shows a change in the analog affinity relative to one peptide chosen as a control, as a logarithm of the ratio of the two respective IC_50_ values. α-Conotoxin PnIA[A10L] has been selected as a control because it has a single substitution present in the majority of other analogs. This scheme is a vivid demonstration of changes in the affinity for all targets brought about by amino acid substitutions.

Radioligand analysis data allowed the following conclusions. Introduction of a charged residue (Arg or Asp) into positions 5 or 7 in most cases drastically decreases the affinity to all targets. The exception is PnIA[L5R, A10L], which was reported to have a 10-fold higher affinity for *L. stagnalis* AChBP than PnIA[A10L] [24], but in our case had only a 10% increase. We are inclined to explain this discrepancy by the essentially different procedures of displacement radioligand assay (see Experimental section).

Introduction of the same substitution may result in different effects on distinct targets. For example, a double “mutation” [L5D, P7R] did not affect the affinity for *A. californica* AChBP, but decreased manifold the affinity for *L. stagnalis* AChBP and α7 nAChR. The reversed substitution [L5R, P7D] decreases the affinity for *A. californica* AChBP as well.

The computer assessment of introducing the positively charged amino acid residue in position 14 was more realistic. This additional “mutation” in some cases (PnIA[L5D, P7R, A10L, D14R]) increases the affinity for *L. stagnalis* AChBP and α7 nAChR, but does not affect the affinity for *A. californica* AChBP, which is in excellent agreement with our earlier data on the activity of the PnIA[A10L, D14K] analog (see Figure 2 and [1]). A positive result of computer modeling application is the addition to this analog (which was effective but not selective among the three mentioned targets) of a more potent α7 nAChR ligand, namely PnIA[L5R, A10L, D14R]. On the other hand, a PnIA[L5H] analog proved to have a high affinity and selectivity for *A. californica* AChBP, with IC_50_ 3.1 ± 0.4 nM (Table 2). For the above-mentioned analog with three substitutions, IC_50_ varied in different experiments from 340 ± 40 to 670 ± 50 nM.

These values need to be commented on because the [^125^I]-αBgt displacement by α-conotoxins from α7 nAChR in the GH_4_C_1_ cells usually is detected at micromolar concentrations. However, electrophysiology experiments, assessing the blocking effects on currents in α7 nAChRs, gave an IC_50_ range of 13–260 nM for PnIA[A10L] and PnIA[A10L, D14K] [1,19]. Thus, this difference depends on the methods used. However, there are possibilities for improvement of radioligand assay results. Recalculation of the IC_50_ values into K_i_s could give more appropriate affinity parameters, especially for the targets with multiple binding sites. The second possibility, is using a radioligand which would be more appropriate in structure and kinetic characteristics for testing the competition of α-conotoxins. Almost irreversible binding of [^125^I]-αBgt makes the detection of competition for α-conotoxins quite difficult, having fast dissociation rates from the target surface. That is why it is desirable to assess binding of novel α-conotoxins in displacement not only of [^125^I]-αBgt, but of the radioactive α-conotoxins as well. We have earlier used such an approach for a number of α-conotoxins acting on the muscle-type nAChR [27,39] and in the present work: the three above-mentioned α-conotoxin analogs, (PnIA[A10L, D14K], PnIA[L5H] and PnIA[L5R, A10L, D14R]), were radioiodinated.

### 2.4. Experiments with Radioactive Forms of α-Conotoxin Analogs

#### 2.4.1. Preparation of Radioactive Derivatives

**Figure 3:**
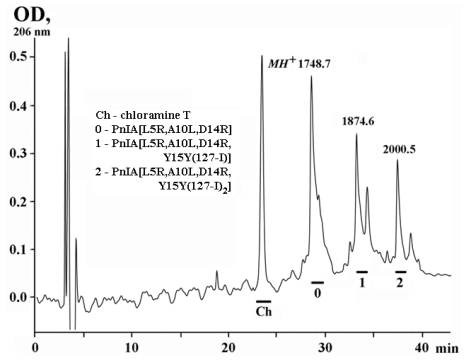
HPLC profile for the products of the [^127^I]iodination reaction of PnIA[L5R, A10L, D14R] analog. The peaks of oxidizer (chloramine T), non-modified analog and mono- and di-iodinated derivatives are marked with indicated molecular masses (*MH*^+^) measured by MALDI mass-spectrometry.

Chemical modification of PnIA[A10L, D14K], PnIA[L5H] and PnIA[L5R, A10L, D14R] in the presence of chloramine T gave their [^125^I]-labeled derivatives. Iodine incorporates mostly to the Tyr15 phenol group, and for each iodinated peptide both mono- and di-iodinated derivatives were formed and then separated by reverse-phase HPLC. Figure 3 shows the separation of the iodinated derivatives after modification of PnIA[L5R, A10L, D14R] with nonradioactive isotope [^127^I]. Iodinated derivatives of the other two α-conotoxins were prepared and separated in a similar way. The structures of all iodinated derivatives were confirmed by MALDI-TOF mass-spectrometry (found masses *MH*^+^ shown in Figure 3 are exactly the same as calculated).

Using this scheme and [^125^I] we prepared the PnIA [A10L, D14K] and PnIA [L5H] iodinated derivatives with the specific radioactivity of 2000 and 4000 Ci/mmol for mono-and di-iodinaned compounds, respectively. Taking into account the acting concentrations of PnIA [L5R, A10L, D14R] (see Table 2), its iodination was done with a mixture of [^125^I] and [^127^I] isotopes resulting in mono and di-iodinated products with the specific radioactivity ~5 and 10 Ci/mmol. In further experiments only mono-iodinated derivatives were used.

#### 2.4.2. Direct Radioligand Assay

**Figure 4:**
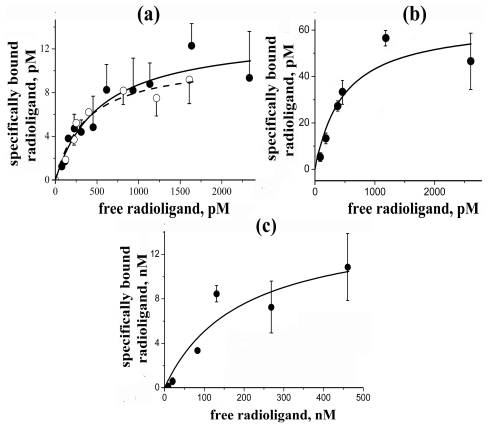
Specific binding curves of [^125^I]-labeled derivatives of (**a**) PnIA[A10L, D14K], (**b**) PnIA[L5H], and (**c**) PnIA[L5R, A10L, D14R] to AChBPs (from *L. stagnalis* (●) or *A. californica* (○)), *A. californica* AChBP and human α7 nAChR, respectively. Data from just one experiment (with mean ± s.e. of duplicate or triplicate for each point (*n* = 2 or 3)) are represented; the respective *K*_D_ values were (**a**) 0.53 ± 0.17 and 0.36 ± 0.10 nM; (**b**) 0.48 ± 0.2 nM; and (**c**) 190 ± 130 nM.

In the direct radioligand assay, [^125^I]-PnIA[A10L, D14K] revealed a capacity to bind with both AChBPs with sub-nanomolar affinity (Figure 4a), *K*_D_ being 0.53 ± 0.17 nM and 0.36 ± 0.10 nM for *L. stagnalis* and *A. californica* AChBPs, respectively. [^125^I]-PnIA[L5H] (Figure 4b) showed similar high affinity for *A. californica* AChBP (*K*_D_ 0.48 ± 0.20 nM) and, in addition, demonstrated a high selectivity without interacting under the same conditions with *L. stagnalis* AChBP or human α7 nAChR.

Iodinated PnIA[L5R, A10L, D14R] showed specific binding to α7 nAChR (Figure 4c), but also had a very high nonspecific binding to the GH_4_C_1_ cells; the reasons for which are unclear. This made difficult accurate determination of the *K*_D_ values which in different experiments varied from 190 ± 130 nM (Figure 4c) to ~1600 ± 800 nM (mean ± s.e.); the averaged value from all experiments being ~900 ± 600 nM (mean ± s.e.m. for *n* = 6).

In all cases, the measured *K*_D_ values are in good agreement with the IC_50_ values found for the starting non-iodinated α-conotoxins in competition with [^125^I]-αBgt. It shows the applicability of radioiodinated α-conotoxins both for labeling the respective targets and for analysis of binding capacities for novel α-conotoxins. To verify the latter, we analyzed binding of several compounds in competition with [^125^I]-PnIA[L5H] and [^125^I]-PnIA[L5R, A10L, D14R] and compared results to same in competition with [^125^I]-αBgt.

#### 2.4.3. Competition Radioligand Assay with [^125^I]-Labeled Derivatives of α-Conotoxins

**Figure 5:**
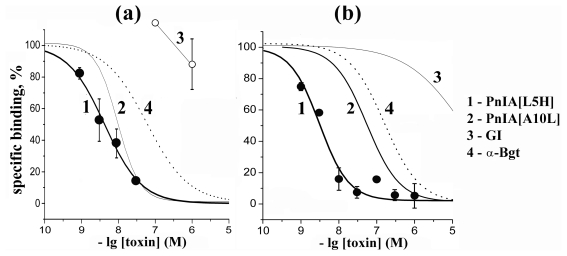
Inhibition of [^125^I]-labeled derivatives of (**a**) PnIA[L5H], and (**b**) α-bungarotoxin (αBgt) binding to *A. californica* AChBP with indicated α-conotoxins and αBgt: (**1**) α-conotoxin PnIA[L5H] (filled circles, thick line), (**2**) α-conotoxin PnIA[A10L] (thin line), (**3**) α-conotoxin GI (open circles, thin line) and (**4**) αBgt (dot line). The curves were calculated from the data of the means of four or five (*n* = 4 or 5) independent experiments in duplicate for each point. For simplicity the mean ± s.e.m. are presented only for PnIA[L5H] analog. The respective IC_50_ values are listed in Table 3.

The results for [^125^I]-PnIA[L5H] and [^125^I]-αBgt on *A. californica* AChBP are shown in Figure 5a,b and the calculated IC_50_ values are given in Table 3. For α-conotoxin PnIA[L5H], these values practically coincide, while all the other compounds in competition with radioactive α-conotoxin show 5–30-fold higher affinity. αBgt in competition with the radioactive α-conotoxin had less activity against *A. californica* AChBP, as earlier found in competition with [^125^I]-αBgt [1]. α-Conotoxin GI acting on muscle-type nAChR was inactive in both cases. It should be emphasized that using [^125^I]-labeled α-conotoxin instead of [^125^I]-αBgt gives better IC_50_ values for tested α-conotoxins which, because of their closer relation to the α-conotoxin radioligand, better reflects their affinity for the respective target.

**Figure 6:**
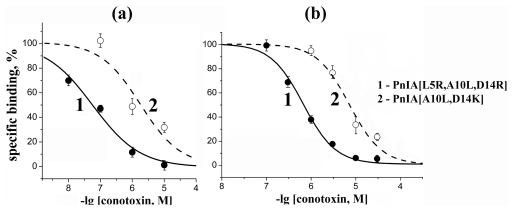
Inhibition of [^125^I]-labeled derivatives of (**a**) PnIA[L5R, A10L, D14R], and (**b**) αBgt binding to human α7 nAChR with (**1**) PnIA[L5R, A10L, D14R] (filled circles, thick line) and (**2**) PnIA[A10L, D14K] (open circles, dashed line). The curves were calculated from the data of the mean of three (*n* = 3) or five (*n* = 5) independent experiments in duplicate for each point in the first or second case, respectively. The calculated IC_50_ values are listed in Table 4.

Similar results were obtained for [^125^I]-PnIA[L5R, A10L, D14R] and [^125^I]-αBgt binding to human α7 nAChR in the GH_4_C_1_ cell line. Here we compared the most efficient ligands in our series acting on this receptor (Figure 6 and Table 4). The ratio of their affinities was the same in both cases, while the IC_50_ values were considerably lower when measured with radioactive α-conotoxin PnIA analog.

Choosing which α-conotoxin analogs to synthesize, we relied upon computer modeling and simple docking algorithms [1,27–29], without performing molecular dynamics. As shown in this paper, only three out of 14 chosen compounds gave results which agreed with the predictions. We have earlier described that docking predictions for α-conotoxin ImII agreed only in part with the experimental results obtained for the AChBPs, muscle and neuronal nAChRs with the aid of radioligand analysis, electrophysiology and surface plasmon resonance [40]. One of the reasons may be that amino acid substitutions in α-conotoxins, or mutations in the receptor binding site of a particular receptor subtype, may induce a change in the ligand orientation. This means that some of the interactions which were expected to take place as a result of the introduced receptor mutations or changes in the ligand structure, did not take place. Indeed, a small change in the orientation of the α-conotoxins in the binding site of the *A. californica* AChBP was observed when comparing the X-ray structures for complexes of PnIA[A10L, D14K] and α-conotoxin TxIA[A10L] which have very similar chemical and spatial structures [1,24]. It has recently been shown that, according to photo-crosslinking data, bound azido-epibatidine has one orientation in the muscle-type nAChR, and two orientations, differing by 180°, in the α4β2 nAChR [41]. Moreover, recent X-ray structures of the *A. californica* AChBP complexes with d-tubocurarine and strychnine, revealed not only their different orientations in the five binding sites of the same AChBP molecule, but even the presence of two ligands in some of them [42]. These results clearly show that more efficient use of computer modeling is necessary, as well as application of molecular dynamics and other mathematical approaches [42].

In spite of the discussed limitations, the main result of this work is that we have synthesized several α-conotoxins which might be useful both for fundamental research on nAChRs and for practical application. First of all, we should mention radioiodinated α-conotoxins for use in binding assays with distinct AChBPs. Among them there are the compounds which have *K*_D_ ~ 0.3–0.5 nM in binding to AChBPs, which is among the highest affinities known for α-conotoxins. The affinity of radioiodinated α-conotoxin PnIA[L5R, A10L, D14R] is also relatively high for α7 nAChR, but a high level of nonspecific binding hampers practical application of this analog. However, what is more important for radioiodinated α-conotoxins is not the affinity as such, but their usefulness in assessing the activity of other α-conotoxins in competition tests. Tables 3 and 4 show that for the majority of analyzed α-conotoxins, their IC_50_ values, measured in competition with radioactive α-conotoxins, are about 10-times lower than those found in competition with [^125^I]-αBgt. Thus, a combination of AChBPs with radioiodinated α-conotoxins appears to be very promising for testing new α-conotoxins, because it has distinct advantages over [^125^I]-αBgt with its virtually irreversible binding.

## 3. Experimental Section

### 3.1. Computer Modeling

In this study we used partial comparative modeling under MODELLER 7v7 program [43]. The model of α7 nAChR was built using Swiss-Prot Server [44]. The programs DOCK [45], HEX [46], AUTODOCK [47], HADDOCK [48] were used for intermolecular docking simulations and energy calculations. Solutions that contradicted known pair-wise interactions were rejected. The detailed description for computer modeling and docking was published by us elsewhere [1,29].

### 3.2. Peptide Synthesis

Solid-phase peptide synthesis was used for preparation of all α-conotoxin PnIA analogs. We applied two different approaches. The first one was the synthesis using the same trityl protection of the cysteine thiol groups followed by simultaneous deprotection and closing the disulfides. In the cases of formation of the second Cys-Cys isomer (in amounts of more than 15%), we carried out the synthesis with the use of the different protection groups for respective cysteine pairs and selective formation of the disulfides. The first protocol was utilized many times for preparation of various α-conotoxins and their analogs and was described in detail in [49]; the second one was given in full in [27], where it was applied for PnIA[A10L, D14K] synthesis.

### 3.3. Iodination

Before preparation of radiolabeled derivatives of PnIA[A10L, D14K], PnIA[L5H] and PnIA[L5R, A10L, D14R] we carried out a series of experiments with nonradioactive [^127^I]-isotope to optimize the reaction and purification conditions, as well as to confirm the structures of iodinated products by MALDI TOF mass spectrometry. Peptides (3–5 nmoles) dissolved in 15 μL of 125 mM sodium phosphate buffer, pH 7.2, were incubated 15 min at room temperature with 3–5 nmoles of NaI (in 4 μL of the same buffer) and 4–10-fold molar excess of chloramine T (solved in 1 μL of water). Reaction products were separated by reverse-phase HPLC in aqueous gradient of acetonitrile containing 0.1% trifluoroacetic acid. The best separation of the reaction products were obtained: for PnIA[A10L, D14K] on a column Nucleosil C_18_ (250 × 4.6 mm) at a rate of 1 mL/min in acetonitrile gradient 10–50% for 40 min; for PnIA[L5H] and PnIA[L5R, A10L, D14R] (see Figure 2) on a column Reprosil C_18_ AQ (5 μm; 150 × 4 mm) at a rate of 0.5 mL/min and acetonitrile gradient 15–35% for 40 min and 10–40% for 60 min, respectively. Purified peaks were analyzed by MALDI TOF mass spectrometry resulting in identification of non-modified, mono- and di-[^127^I]-iodinated compounds.

The similar protocols (but with different amounts of reaction components) were applied for preparation of radioactive derivatives using Na[^125^I] solution:

120 pmoles PnIA[A10L,D14K]+90 pmoles Na[I125]+1000 pmoles chloramine T

or

80 pmoles PnIA[L5H]+65 pmoles Na[I125]+4.4 nmoles chloramine T

For PnIA[L5R, A10L, D14R] analog we used the isotopic mixture of Na[^125^I] and Na[^127^I] at the ratio of 1:333, so the reaction protocol was:

9.6 nmoles PnIA[L5R,A10L,D14R]+10 nmoles Na[I125+127]+44 nmoles chloramine T

Reaction products were separated by HPLC under above-mentioned conditions and collected in 0.5 min-fractions. The aliquots of all fractions were counted on γ-counter and mono- and di-[^125^I]iodinated derivatives (with approximate specific radioactivity of 2000 and 4000 Ci/mmol) of PnIA[A10L, D14K] and PnIA[L5H] analogs as well as PnIA[L5R, A10L, D14R] analog (with approximate specific radioactivity of 6 and 12 Ci/mmol) were collected in respective united samples. These samples were evaporated (approximately to 50% of initial volume) to remove acetonitrile and were kept at 4 °C in 50 mM Tris-HC1 buffer, pH 7.5, containing 0.1 mg/mL BSA.

We only used mono-[^125^I]iodinated derivatives of all analogs in our studies.

### 3.4. Radioligand Assay

In competition experiments with [^125^I]-αBgt, all synthesized α-conotoxin PnIA analogs (concentration range for concrete peptide was varied inside 1–100,000 nM) were pre-incubated 2.0–2.5 h at room temperature with the *L. stagnalis* or *A. californica* AChBPs (final concentrations of 2.4 and 140 nM, respectively) in 50 μL of buffer A (phosphate-buffered saline, 0.7 mg/mL of bovine serum albumin, 0.05% Tween 20, pH 7.5) or with the GH_4_C_1_ cells (final 6.5 μg of total protein with 0.4 nM of toxin-binding sites) in 50 μL of buffer B (20 mM Tris–HCl buffer, 1 mg/mL of bovine serum albumin, pH 8.0). After that, [^125^I]-αBgt was added to *L. stagnalis* AChBP, *A. californica* AChBP or GH_4_C_1_ cells at final concentration 0.1, 0.3 or 0.2 nM and the mixtures were additionally incubated for 30 min, 30 min or 5 min, respectively. The specific binding was determined by rapid filtration on double DE-81 filters (Whatman) pre-soaked in buffer A (for AChBPs) or on GF/F filters (Whatman) pre-soaked in 0.25% polyethylenimine (for GH_4_C_1_ cells) and the unbound radioactivity was removed from the filters by washes (3 × 3 mL) with the buffers A and B, respectively. Non-specific binding was determined in all cases in the presence of 2 μM α-cobratoxin (2.0–2.5 h pre-incubation).

In competition experiments with [^125^I]-PnIA[L5H], selected α-conotoxin PnIA analogs or α-Bgt (concentration range for concrete compound varied within 1–1000 nM) were pre-incubated 1.5 h at room temperature with the *A. californica* AChBP (final concentration of 2.3 nM) in 50 μL of buffer A followed by adding radioligand (final 0.6–1.0 nM) and additional incubation during 1 h. Non-specific binding was determined in the presence of 3.8 μM α-cobratoxin. The filtration was performed as mentioned above for experiments with [^125^I]-αBgt on the AChBPs.

In competition experiments with [^125^I]-PnIA[L5R, A10L, D14R], two analogs—PnIA[A10L, D14K] and PnIA[L5R, A10L, D14R] (from 10 to 30,000 nM)—were incubated 1.5 h at room temperature and permanent shaking with GH_4_C_1_ cells (0.4 nM of toxin-binding sites of α7 nAChR) in 50 μL of buffer B. After that, radioligand (final concentration of 180 nM) was added and the reaction mixture was incubated additional 1.5 h under the same conditions. Non-specific binding was determined by 1.5 h pre-incubation with 25 μM α-cobratoxin. The filtration was performed as mentioned above for experiments with [^125^I]-αBgt on the GH_4_C_1_ cells.

Competition data analyses were fit using ORIGIN 7.5 (OriginLab Corporation, Northampton, MA, USA) to a one-site dose-response curve by Equation: % response = 100/{1 + ([toxin]/IC_50_)*^n^*^_H_^}, where IC_50_ is the concentration at which 50% of the sites are inhibited and *n*_H_ is the Hill coefficient.

Equilibrium binding of [^125^I]-PnIA[A10L, D14K] with both AChBPs (final concentration 1.2 nM) was carried out in 50 μL of buffer A at room temperature. Various concentrations of radioligand (0.05–2.5 nM) were incubated with proteins for 30 min. Non-specific binding was determined in the presence of α-cobratoxin at a 500-fold molar excess over the radioligand (1 h pre-incubation). The filtration was performed as mentioned above for AChBPs.

The similar protocol was applied for equilibrium binding of [^125^I]-PnIA[L5H] with *A. californica* AChBP. We incubated in this case 0.1–2.8 nM of radioligand with 2.3 nM of protein during 2 h at room temperature. Non-specific binding was determined by 1 h pre-incubation with 3.8 μM α-cobratoxin.

Equilibrium binding of [^125^I]-PnIA[L5R, A10L, D14R] with human α7 nAChR transfected in rat GH_4_C_1_ cell line was carried out in 50 μL of buffer B at room temperature during 1.5 h with permanent shaking. Various concentrations of radioligand (6–1600 nM) were incubated with 0.4 nM of toxin-binding sites of α7 nAChR. Non-specific binding was determined by 1.5 h pre-incubation with 25 μM α-cobratoxin. The specific binding was determined by rapid filtration on GF/F or GF/C filters pre-soaked in 0.25% polyethylenimine and the unbound radioactivity was removed from the filters by washes (3 × 3 mL) with the buffer B.

Equilibrium binding data were fit using ORIGIN 7.5 to a one-site model according to Equation: *B*(*x*) = *B*_max_/(1 + *K*_D_/*x*), where *B*(*x*) is the radioligand specifically bound at a free concentration *x* (determined by subtraction of the amount of bound and adsorbed radioligand from the total amount added to incubation mixture), *B*_max_ is the maximal specific bound radioligand, and *K*_D_ is the dissociation constant.

## 4. Conclusions

Our work demonstrates that the available X-ray structures of AChBP complexes with α-conotoxins are a good starting point for design and subsequent synthesis of novel α-conotoxins having higher affinity and desired selectivity. In fact, we prepared several analogs of higher affinity, better discriminating the AChBPs from *A. californica* and *L. stagnalis*. Our achievements are more modest for α-conotoxins targeting the α7 nAChR, apparently because no X-ray structure is available either for this receptor or for its extracellular ligand-binding domain, and the used computer modeling and docking approaches were insufficient to take into account possible multiple orientations of bound α-conotoxin analogs. However, by preparing radioiodinated derivatives of several synthesized analogs, we demonstrated that the competition with such radioactive α-conotoxins is a better way to test new α-conotoxins than the radioligand analysis with radioiodinated α-bungarotoxin.

## Figures and Tables

**Table 1:** Structures and molecular masses of synthesized α-conotoxin PnIA analogs with amino acid substitutions suggested by computer modeling. Positively charged substitutions are marked in red; negatively charged—in blue; aliphatic or aromatic substitutions—in gray. PnIA[A10L], PnIA[A10L, D14K] and PnIA[L5R, A10L] were described earlier [1,18,19,24]. For comparison, the sequence of wild type α-conotoxin PnIA is included.

Analogs of α-conotoxin PnIA	Sequences and mutations	Molecular masses	
		Measured (*MH*^+^)	Calculated
PnIA	**GCCSLPPCAANNPDYC-NH_2_**	-	-
PnIA[L5H]	**GCCSHPPCAANNPDYC-NH_2_**	**1646.6**	**1645.6**
PnIA[L5H, D14R]	**GCCSHPPCAANNPRYC-NH_2_**	**1687.3**	**1686.6**
PnIA[A10L]	**GCCSLPPCALNNPDYC-NH_2_**	**1664.7**	**1663.7**
PnIA[L5D, A10L]	**GCCSDPPCALNNPDYC-NH_2_**	**1666.7**	**1666.4**
PnIA[L5R, A10L]	**GCCSRPPCALNNPDYC-NH_2_**	**1707.6**	**1706.6**
PnIA[P7D, A10L]	**GCCSLPDCALNNPDYC-NH_2_**	**1682.4**	**1681.6**
PnIA[P7R, A10L]	**GCCSLPRCALNNPDYC-NH_2_**	**1723.7**	**1722.9**
PnIA[A10L, D14K]	**GCCSLPPCALNNPKYC-NH_2_**	**1677.6**	**1676.8**
PnIA[L5D, P7R, A10L]	**GCCSDPRCALNNPDYC-NH_2_**	**1725.7**	**1724.9**
PnIA[L5D, P7R, A10V]	**GCCSDPRCAVNNPDYC-NH_2_**	**1711.6**	**1710.8**
PnIA[L5R, P7D, A10L]	**GCCSRPDCALNNPDYC-NH_2_**	**1725.4**	**1724.6**
PnIA[L5R, A10L, D14R]	**GCCSRPPCALNNPRYC-NH_2_**	**1748.5**	**1747.7**
PnIA[L5D, P7R, A10L, D14R]	**GCCSDPRCALNNPRYC-NH_2_**	**1766.7**	**1766.0**
PnIA[L5R, P7D, A10L, D14R]	**GCCSRPDCALNNPRYC-NH_2_**	**1766.5**	**1765.7**
PnIA[L5Y, P6R, P7R, A10L, D14R, Y15W]	**GCCSYRRCALNNPRWC-NH_2_**	**1896.8**	**1895.8**

**Table 2:** Activity of α-conotoxin PnIA analogs tested in competition binding assays. All listed peptides were tested in competition with [^125^I]-labeled α-bungarotoxin ([^125^I]-αBgt) for binding to AChBPs and human α7 nAChR. The presented IC_50_ values (in nM) and Hill coefficients (*n*_H_) were calculated using ORIGIN 7.5 with the mean ± s.e.m. of duplicate data obtained in two or three (*n* = 2 or 3) independent experiments for most analogs. More data were collected for analogs PnIA[L5H], [L5R, A10L, D14R], [A10L], [A10L, D14K] and [L5R, A10L] with *n* = 5, 5, 5, 6 and 8, respectively.

Mutations in PnIA	IC_50_ in nM and Hill slopes (*n*_H_) in [^125^I]-αBgt displacement from					
	*L. stagnalis* AChBP		*A. californica* AChBP		human α7 nAChR	
[L5H]	220 ± 80	(0.71 ± 0.06)	3.1 ± 0.4	(1.13 ± 0.15)	26,000 ± 1000	(1.15 ± 0.05)
[L5H, D14R]	2900 ± 100	(1.18 ± 0.05)	1400 ± 100	(1.31 ± 0.09)	21,000 ± 1000	(1.01 ± 0.05)
[A10L]	200 ± 40	(0.89 ± 0.13)	55 ± 12	(1.18 ± 0.24)	14,000 ± 1000	(0.73 ± 0.04)
[L5D, A10L]	35,000 ± 3000	(1.45 ± 0.20)	5200 ± 1900	(1.03 ± 0.17)	>100,000	(−)
[L5R, A10L]	180 ± 20	(1.10 ± 0.10)	305 ± 19	(1.51 ± 0.25)	12,000 ± 2000	(1.03 ± 0.11)
[P7D, A10L]	>>100,000	(−)	63,000 ± 11,000	(0.79 ± 0.11)	>>100,000	(−)
[P7R, A10L]	>100,000	(−)	1250 ± 300	(1.00 ± 0.23)	>>100,000	(−)
[A10L, D14K]	8.2 ± 1.2	(1.01 ± 0.11)	47 ± 9	(0.77 ± 0.12)	7200 ± 700	(1.20 ± 0.11)
[L5D, P7R, A10L]	38,000 ± 8000	(0.69 ± 0.09)	51 ± 11	(1.38 ± 0.31)	>>100,000	(−)
[L5D, P7R, A10V]	6400 ± 1300	(1.04 ± 0.22)	45 ± 11	(1.35 ± 0.42)	>100,000	(−)
[L5R, P7D, A10L]	56,000 ± 2000	(1.48 ± 0.15)	28,000 ± 4000	(0.91 ± 0.09)	>>100,000	(−)
[L5R, A10L, D14R]	430 ± 90	(1.20 ± 0.30)	1400 ± 100	(1.27 ± 0.16)	670 ± 50	(1.20 ± 0.19)
[L5D, P7R, A10L, D14R]	1200 ± 250	(0.73 ± 0.10)	46 ± 8	(1.44 ± 0.28)	23,000 ± 1000	(0.91 ± 0.13)
[L5R, P7D, A10L, D14R]	4100 ± 200	(1.25 ± 0.07)	3200 ± 100	(0.73 ± 0.12)	72,000 ± 5000	(0.84 ± 0.06)
[L5Y, P6R, P7R, A10L, D14R, Y15W]	10,000 ± 1000	(1.09 ± 0.05)	20,000 ± 2000	(1.26 ± 0.18)	19,000 ± 1000	(1.20 ± 0.26)

**Table 3:** Potency (presented as IC_50_ values in nM) and Hill coefficients (*n*_H_) of some α-conotoxins and α-conotoxin PnIA analogs, tested in competition with [^125^I]-labeled PnIA[L5H] or [^125^I]-αBgt for binding to *A. californica* AChBP (see respective inhibition curves in Figure 5), calculated using ORIGIN 7.5 with the mean ± s.e.m. of duplicate data obtained in *n* = 4 or 5 independent experiments.

Compound	IC_50_ in nM and Hill slopes (*n*_H_) in competition with			
	[^125^I]-PnIA[L5H]		[^125^I]-αBgt	
PnIA[L5H]	4.2 ± 0.3	(0.87 ± 0.05)	3.1 ± 0.4	(1.13 ± 0.15)
PnIA[A10L]	9.5 ± 1.1	(1.40 ± 0.20)	36–55 *	
PnIA[A10L, D14K]	2.2 ± 0.6	(1.42 ± 0.49)	28–47 *	
α-conotoxin ImI	0.84 ± 0.15	(1.37 ± 0.29)	33 ± 5 **	
α-conotoxin GI	>>1000	(−)	25,500 ± 6300 **	
α-bungarotoxin (αBgt)	60 ± 10	(0.77 ± 0.08)	130 ± 20 **	

* dispersion of the mean IC_50_ values in this study and experiments carried out by us earlier [1];

** data from [1].

**Table 4:** Potency (presented as the IC_50_ values in nM) and Hill coefficients (*n*_H_) of PnIA[L5R, A10L, D14R] and PnIA[A10L, D14K] analogs, tested in competition with [^125^I]-labeled PnIA[L5R, A10L, D14R] or [^125^I]-αBgt for binding to human α7 nAChR (see respective inhibition curves shown in Figure 6). The data were calculated using ORIGIN 7.5 with the mean ± s.e.m. of duplicate data obtained in *n* = 3 or 5 independent experiments, respectively.

Compound	IC_50_ in nM and Hill slopes (*n*_H_) in competition with			
	[^125^I]-PnIA[L5R, A10L, D14R]		[^125^I]-αBgt	
PnIA[L5R, A10L, D14R]	60 ± 7	(0.65 ± 0.05)	670 ± 50	(1.20 ± 0.19)
PnIA[A10L, D14K]	1800 ± 600	(0.75 ± 0.14)	7200 ± 700	(1.20 ± 0.11)
